# Intravitreal Therapy for Diabetic Macular Edema: An Update

**DOI:** 10.1155/2021/6654168

**Published:** 2021-02-23

**Authors:** Claudio Furino, Francesco Boscia, Michele Reibaldi, Giovanni Alessio

**Affiliations:** ^1^Department of Medical Science, Neuroscience and Sense Organs, Eye Clinic, University of Bari, Azienda Ospedaliero-Universitaria Policlinico Bari, Bari, Italy; ^2^Department of Surgical Sciences, Eye Clinic, University of Torino, Torino, Italy

## Abstract

Diabetic macular edema (DME) represents a prevalent and disabling eye condition. Despite that DME represents a sight-threatening condition, it is also among the most accessible to treatment. Many different treatment options including photocoagulation, intravitreal medical treatment (either vascular endothelial growth factor inhibitors or corticosteroids therapies), and surgical removal are currently available. Although laser has been considered as the gold standard for many years, over the past several years vascular endothelial growth factor inhibitors (anti-VEGFs) have become first-line therapy. However, many patients do not adequately respond to them. With the development of sustained-release corticosteroid devices, steroids have gained a presence in the management of the DME. We review and update the role of anti-VEGF and intravitreal sustained-release corticosteroid management of DME. According to the currently available scientific evidence, the choice of one anti-VEGF over another critically depends on the baseline best-corrected visual acuity (BCVA). While aflibercept may be the drug of choice in low baseline BCVA, the three anti-VEGFs (bevacizumab, ranibizumab, and aflibercept) provided similar functional outcomes when the baseline BCVA was higher. DEX implants are a valuable option for treating DME, although they are usually seen as a second choice, particularly in those eyes that have an insufficient response to anti-VEGF. The new evidence suggested that, in eyes that did not adequately respond to anti-VEGF, switching to a DEX implant at the time to 3 monthly anti-VEGF injections provided better functional outcomes.

## 1. Introduction

Because the prevalence of diabetes is rising, the relevance of diabetic eye disease increases [1, 2]. In Europe, it was estimated that approximately 6.4 million people are currently affected by any diabetic eye disease and 8.6 million people will be affected in 2050 [3]. In the year 2020, moderate to severe visual impairment due to diabetic retinopathy has been estimated in 4.06% (Western Europe), 4.77% (Asia-Pacific, high income), and 4.99% (United States, high income) [2].

Diabetic macular edema (DME) is a chronic, multifactorial, and sight-threatening condition that critically impacts patients' quality of life [4, 5].

The prevalence of any DME in Europe was 3.7% and its pooled mean annual incidence in type 2 diabetes patients was 0.4% [3].

Despite that DME represents a sight-threatening condition, it is also among the most accessible to treatment. Many different treatment options including laser photocoagulation, intravitreal medical treatment (either vascular endothelial growth factor inhibitors or corticosteroids therapies), and pars plana vitrectomy [6] are currently available.

A retrospective analysis, conducted on a population of 13,410 treatment-naïve DME patients, has found that the treatment patterns within 28 days of initial DME diagnosis were as follows: observation in 9,990 (74.5%) patients, vascular endothelial growth factor inhibitors (anti-VEGFs) in 2,086 (15.6%) patients, laser in 1,133 (8.4%) patients, corticosteroids in 133 (1.0%) patients, and combined treatment in 68 (0.5%) patients [7].

The main purpose of this paper was to review the role of anti-VEGF and intravitreal sustained-release corticosteroid devices for treating patients with DME. Additionally, this paper is also going to evaluate the current evidence about the convenience of switching to intravitreal dexamethasone implant in those patients with suboptimal response to anti-VEGF therapies.

## 2. Pathophysiology

A complete review of the pathophysiologic mechanisms in DME is beyond the scope of this paper.

Macular edema (ME) is defined as an abnormal increase of fluid volume in the macula [8]. The etiology and pathogenesis of DME are multifactorial and result from multiple and intricate mechanisms. Although hyperglycemia is the main risk factor for diabetic retinopathy, different factors including hypoxia, impaired blood flow, retinal ischemia, and inflammation are also associated with DME [8, 9].

Different molecules such as interleukin-6 (IL-6), IL-8, IL-1B, vascular endothelial growth factor (VEGF), and tumor necrosis factor-*α* (TNF-*α* ) are upregulated in eyes with DME [9, 10]. These mechanisms cause a disruption of the blood-retinal barrier that not only leads to the accumulation of subretinal and intraretinal fluid but also stimulates the expression of adhesion molecules that facilitate the adhesion capacity of inflammatory cells [9, 10].

Recent investigations have shown that chronic hyperglycemia induces oxidative stress and inflammation in the retina, which constitutes early processes in the development of DME [8, 11]. Increased inflammation is associated with capillary nonperfusion and breakdown of the blood-retina barrier [8, 10, 11]. Inflammation is not only a consequence of barrier dysfunction but also an early local mechanism contributing to barrier alteration and leukostasis [8, 10, 11].

**Figure 1:**
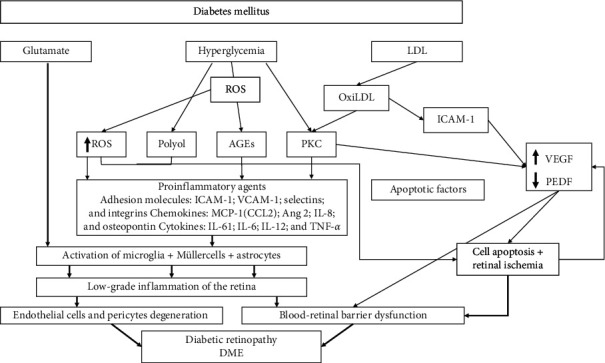
An overview of the different pathways involved in the development of diabetic macular edema (adapted from Daruich et al. [8] and Romero-Aroca et al. [9]). LDL: low-density lipoprotein; ROS: reactive oxidative species; Oxi: oxidized; AGEs: advanced glycation end-products; PKC: protein kinase C; ICAM-1: inflammatory intercellular adhesion molecule-1; VEGF: vascular endothelial growth factor; VCAM-1: vascular cell adhesion molecule-1; PEDF: pigment epithelium-derived factor; CCL2: chemokine C-C motif ligand 2; Ang-2: angiopoietin-2; IL: interleukin; TNF: tumor necrosis factor; DME: diabetic macular edema.

Inflammatory cytokines, which mediate vascular permeability, such as tumor necrosis factors alpha and beta, alpha 4 integrin, nitric oxide, and interleukin-1*β* are elevated in DME [8–11] (Figure 1).

## 3. Treatment Strategies of DME

Although laser treatment has been considered as the gold standard for many years [12, 13], according to the European Society of Retina Specialists (EURETINA) guidelines, focal/grid laser is now reserved mostly for non-center-involving DME [6].

Currently, anti-VEGF agents are considered the first line of treatment in center-involving DME; however, all the large clinical trials have shown that only 33–45% of DME patients on anti-VEGF agents show 3 lines or more of visual improvement [6, 14, 15]. The inadequate response to anti-VEGF observed in many patients speaks in favor of the presence of other factors beyond VEGF, such as inflammation, which is not targeted by the anti-VEGF drugs. There is, therefore, a need for supplemental treatments that might improve visual acuity in eyes with persistent edema despite anti-VEGF therapy.

**Figure 2:**
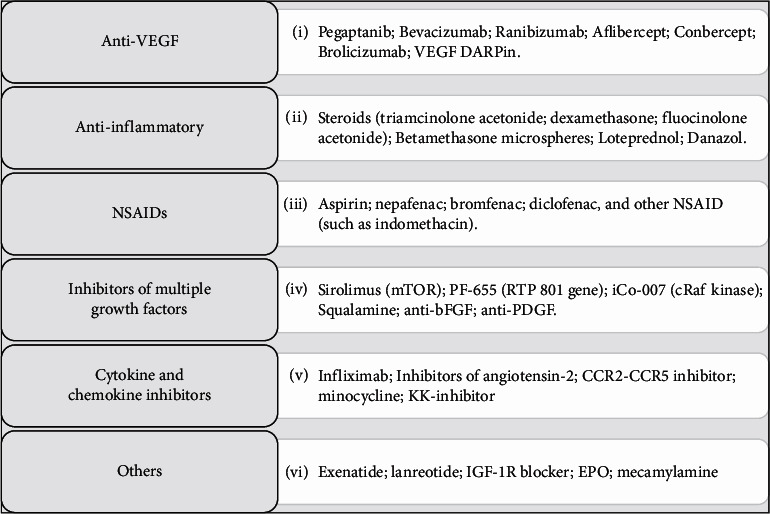
Overview of the medical treatment options for diabetic macular edema (adapted from Urias et al. [15]). NSAIDS: nonsteroidal anti-inflammatory drug; VEGF: vascular endothelial growth factor; DARPin: designed ankyrin repeat protein; FGF: fibroblast growth factor beta; PDGF: platelet-derived growth factor; CCR: chemokine receptor; IGF-1: insulin-like growth factor-1; EPO: erythropoietin.

An overview of the currently available and future options for treating DME is summarized in Figure 2.

### 3.1. Vascular Endothelial Growth Factor Inhibitors

The introduction of anti-VEGF agents has revolutionized the medical management of DME. Under the umbrella of the term “anti-VEGF,” there are several different molecules that can be classified as aptamers (pegaptanib), antibodies to VEGF (bevacizumab), antibody fragments to VEGF (ranibizumab), and fusion proteins, which combine a receptor for VEGF with the constant region of a human immunoglobulin (aflibercept and conbercept) [14, 15].

#### 3.1.1. Pegaptanib

Pegaptanib (Macugen®, Bausch and Lomb, Rochester, NY, USA), developed to bind and block the activity of extracellular VEGF [16, 17], was the first commercially available anti-VEGF drug used to treat DME [17]. Cunningham et al. [17] in a randomized, double-masked, multicenter, dose-ranging, and sham-controlled phase II trial evaluated the efficacy and safety of pegaptanib in the treatment of DME. Three different doses of pegaptanib (0.3 mg, 1.0 mg, and 3.0 mg) were tested and compared to sham injections. Injections were planned at baseline, week 6, and week 12, with additional injections and/or focal photocoagulation as needed for another 18 weeks [17]. At week 36 compared with baseline, visual acuity improvement ≥10 letters occurred in 15 of 44 (34%) patients, 13 of 43 (30%) patients, 6 of 42 (14%) patients, and 4 of 41 (10%) patients in the 0.3 mg, 1.0 mg, 3.0 mg, and sham subgroups, respectively (*p* = 0.003, 0.3 mg versus sham) [17].

Sultan et al. [18] evaluated in a randomized (1 : 1), sham-controlled, multicenter, parallel-group clinical trial the efficacy and safety of intravitreal pegaptanib 0.3 mg versus sham injections. Based on the results of this study, the probability of achieving a visual acuity improvement of ≥10 letters, at week 54 compared with baseline, was significantly greater in the pegaptanib group (odds ratio, 2.38; 95% confidence interval, 1.32–4.30; *p* = 0.0047) [18].

Additionally, the results of a Japanese phase III randomized clinical trial found that the proportion of patients who achieved a visual acuity improvement of ≥10 letters, from baseline to week 24, was significantly greater in the pegaptanib group (20.3%) than in the sham group (5%), *p* = 0.0003) [19].

The results of these randomized clinical trials have been confirmed in several studies conducted in clinical settings [20–22].

Similarly, the results of a retrospective study, which evaluated the effect of intravitreal pegaptanib on the functional and anatomical outcomes, found a significant improvement (from baseline to the last follow-up visit) in mean BCVA and a significant reduction in central macular thickness (CMT) (*p* < 0.001 and *p* < 0.001, respectively) [20].

Rinaldi et al. [21] published in 2012 a longitudinal, interventional, and nonrandomized study that evaluated the efficacy and safety of intravitreal pegaptanib in patients with “clinically significant diabetic macular edema”. The results of this study found a significant reduction in foveal thickness (*p* = 0.0001) and a significant improvement in BCVA (*p* < 0.005), macular sensitivity (*p* < 0.001), and color discrimination (*p* = 0.0001) [21].

Sivaprasad et al. [22], in an open-label, one-year, and noncomparative study, evaluated the safety and tolerability of pegaptanib in DME patients. Four (25%) of 12 patients achieved a visual acuity improvement of ≥ 10 letters and 8 (8.7%) of 46 patients reported treatment-related adverse events [22].

#### 3.1.2. Bevacizumab

Bevacizumab, a humanized monoclonal antibody that inhibits vascular endothelial growth factor (VEGF), was originally developed as a concomitant medication for use in combination with existing metastatic colorectal cancer regimens [23].

Intravitreal injections of bevacizumab have been and currently continue to be widely used as an off-label treatment for neovascular age-related macular degeneration and DME [6, 7, 14, 15].

The first study evaluating the efficacy of bevacizumab (Avastin; Genentech, Inc., South San Francisco, CA) for the treatment of persistent DME was published by Haritoglou et al. [24]. The results of this prospective, consecutive, and noncomparative case series study found a significant improvement in visual acuity (*p* = 0.001) and a significant reduction in CMT (*p* = 0.002) [24].

These findings were confirmed by different small studies [25–28].

The Diabetic Retinopathy Clinical Research Network (DRCR.net) published in 2007 the results of a randomized phase II clinical trial that evaluated the efficacy and safety of intravitreal bevacizumab (either alone or in combination with focal photocoagulation) in DME patients [29]. The results of this study suggested some positive findings associated with the use of bevacizumab. However, this response was similar to that observed in the laser group after more than 3 weeks [29].

The BOLT (bevacizumab or laser therapy) study was a prospective, randomized, masked, single-center, 2-year, and 2-arm clinical trial that compared the effect of repeated intravitreal injections of bevacizumab *versus* (*vs.*) modified Early Treatment of Diabetic Retinopathy Study (ETDRS) macular laser therapy in patients with *persistent clinically significant* DME [30]. The results of this study found that, after 12 months of follow-up, the probability of achieving a visual acuity improvement of ≥10 ETDRS letters was significantly greater in the bevacizumab group than in the laser group (adjusted odds ratio, 5.1; 95% confidence interval, 1.3 to 19.7, *p* = 0.019) [30].

The 2-year outcomes of the BOLT study confirmed the aforementioned 12-month data [30]. At 2 years, the proportion of patients gained ≥10 or ≥15 ETDRS letters was significantly greater in the bevacizumab group than in the laser one (*p* = 0.001 and *p* = 0.004, respectively) [31].

Different randomized clinical trials (RCTs), whose results have been summarized in Table 1, have evaluated the efficacy and safety of intravitreal bevacizumab in DME patients [29–34]. Although, on average, intravitreal bevacizumab has shown a positive impact on DME patients, in many studies it was not superior to other therapies.

The Protocol T was a prospective, randomized, and multicenter clinical trial that compared the efficacy and safety of intravitreal injections of bevacizumab, ranibizumab, and aflibercept for the treatment of DME [35]. Patients included in Protocol T were randomly assigned in a 1 : 1 : 1 ratio to be injected with bevacizumab (1.25 mg), ranibizumab (0.3 mg), or aflibercept (2.0 mg) [35]. The mean visual acuity improvement was significantly greater with aflibercept than with bevacizumab (*p* < 0.001) and ranibizumab (*p* = 0.03). However, this advantage was not considered as clinically relevant because the effect of visual acuity varied according to the baseline visual acuity [35]. As regards central subfield thickness, aflibercept achieved the greatest reduction with 169 ± 138 *μ*m (*p* < 0.001 vs. bevacizumab and *p* = 0.0336 vs. ranibizumab), followed by ranibizumab with 147 ± 134 *μ*m (*p* < 0.001 vs. bevacizumab) and bevacizumab with 101 ± 121 *μ*m. However, similarly to visual acuity, the effect on central subfield thickness varied according to initial visual acuity [35].

The main findings of the Protocol T  are shown in Table 2.

The 2-year Protocol *T* results showed slight changes as compared to the 1-year results. However, as shown in the 1-year results, BCVA improvement varied according to the initial visual acuity [36].

*(1) Safety*. Many ophthalmologists have been and currently are worried about the safety profile of bevacizumab. When used to treat certain cancers, intravenous bevacizumab has been associated with several side effects, including systemic hypertension, proteinuria, and cardiovascular and gastrointestinal complications [37].

The results of a post hoc analysis of the Protocol T found that aflibercept and bevacizumab induced greater decreases in plasma free-VEGF than ranibizumab at 4 weeks [38]. Moreover, at 52 and 104 weeks, a greater decrease was observed in bevacizumab versus ranibizumab [38]. Interestingly, this study did not find any significant relationship between VEGF concentration and the incidence of a heart attack or stroke [28].

The incidence of serious ocular and nonocular adverse events was approximately below 1 per 100 injections for intravitreal bevacizumab [28–36]. Several systemic adverse events have been reported in different studies, including systemic hypertension, cerebrovascular accidents, heart attacks, and death [28–36, 39–41]. Ocular side effects included bacterial endophthalmitis, ocular inflammation (iritis, iridocyclitis, uveitis, or vitreous), tractional retinal detachment, pigmentary epithelial detachment, vitreous hemorrhage, ocular hypertension, and cataract [28–36, 39–41].

According to the European Society of Retina Specialists (EURETINA) guidelines, there are still some issues about the anti-VEGF safety profile of bevacizumab that should be further explored [6].

#### 3.1.3. Ranibizumab

Ranibizumab (Lucentis®, Novartis, Basel, Switzerland) is a fully humanized monoclonal antibody fragment, which binds to multiple variants of VEGF-A [42]. It was originally approved for treating neovascular age-related macular degeneration [6].

The first prospective, randomized, interventional, and multicenter clinical trial comparing the effect of ranibizumab with focal/grid laser or a combination of both in DME was the Ranibizumab for Edema of the mAcula in diabetes (READ-2) study [43]. Patients were randomly assigned in a 1 : 1 : 1 regime to receive ranibizumab 0.5 mg (baseline and months 1, 3, and 5), laser (baseline and month 3, if needed), and a combination of ranibizumab 0.5 mg and laser (baseline and month 3) [43]. The results of this study found that, at month 6, BCVA improvement was significantly greater in those eyes receiving ranibizumab alone (*p* = 0.01).

The RESOLVE was a 12-month, multicenter, sham-controlled, double-masked study designed to evaluate the efficacy and safety of ranibizumab in DME [44]. Study patients were randomized to ranibizumab (0.3 or 0.5 mg; *n* = 51 each) or sham (*n* = 49) [44]. The mean average change in BCVA from month 1 to month 12 was significantly greater with ranibizumab (7.8 letters) than with sham (−0.1 letters) (*p* < 0.0001). Similarly, the mean change in CMT was significantly greater with ranibizumab (−194.2 *μ*m) than with sham (−48.4 *μ*m) (*p* < 0.001) [44]. Regarding the safety profile, there were no significant differences in the incidence of serious and nonserious ocular/nonocular adverse events between ranibizumab and sham. In both arms (without significant differences between them), the most frequently reported adverse events were conjunctival hemorrhage, ocular hypertension, and eye pain [44].

The RESTORE study was a 12-month, multicenter, prospective, randomized, double-masked, and laser-controlled phase III study that evaluated the efficacy (in terms of BCVA improvement) of ranibizumab 0.5 mg + sham laser vs. ranibizumab + laser vs. laser alone [45]. As compared to laser, ranibizumab alone achieved a greater BCVA improvement (*p* < 0.0001) and a greater proportion of gaining ≥15 letters (*p* = 0.0005). CMT reduction was significantly greater with ranibizumab alone than with laser alone (*p* < 0.0001). There were no significant differences in either BCVA improvement or CMT reduction between ranibizumab alone and ranibizumab + laser. Regarding the safety profile, there was no significant association between ranibizumab (alone or in combination) and the incidence of cardiovascular or cerebrovascular events [45].

Based on the RESTORE results, the European Medicines Agency (EMAs) approved in 2011 the use of ranibizumab for the treatment of DME [6]. Interestingly, the dose approved by the EMAs (0.5 mg) differs from those approved by the Food and Drug Administration (0.3 mg) [6].

The RISE and the RIDE are two multicenter, prospective, randomized, and sham injection-controlled studies that evaluated the efficacy and safety of ranibizumab in DME patients [46]. Seven hundred and fifty-nine patients (377 in RISE and 382 in RIDE) were randomized to receive monthly sham injections or intravitreal injections of ranibizumab 0.3 mg or 0.5 mg. The proportion of patients gaining ≥15 letters in RISE was the 18.1 %, 39.2%, and 44.8% in eyes treated with sham, ranibizumab 0.5 mg, and ranibizumab 0.3 mg, respectively (*p* < 0.0001 between sham and 0.3 mg and *p* = 0.0002 between sham and 0.5 mg) [46]. Regarding RIDE, a greater proportion of patients achieved a BCVA improvement ≥15 in the ranibizumab 0.3 mg group (33.6%, *p* < 0.0001 vs. sham) and in the ranibizumab 0.5 mg group (45.7%, *p* < 0.0001 vs. sham) than in the sham-treated group (12.3%). BCVA improvements were paralleled by rapid reductions in CMT. Independently of the study (RISE or RIDE) and the ranibizumab dose (0.3 or 0.5), the mean CMT reduction was significantly greater in ranibizumab groups than in sham groups (*p* < 0.0001 each) [46]. Safety profile showed that serious adverse events were uncommon and included one case of endophthalmitis in RISE and 3 in RIDE and 2 cases of traumatic cataract in RISE and one in RIDE. Systemic adverse events potentially related to systemic VEGF inhibitions occurred in 10.6% and 9.4% of sham-treated patients in RISE and RIDE, respectively, and in 5.6% and 1.9% of ranibizumab-treated eyes (both doses) across the studies [46].

The 3-year follow-up results of the study that evaluated the effect of prompt vs. deferred (≥24 weeks) of laser treatment in eyes receiving intravitreal injections of intravitreal ranibizumab (0.5 mg) suggested that, in eyes with DME, deferring laser ≥ 24 weeks provided better functional outcomes than prompt laser treatment [47]. However, this finding might be justified by the fact that eyes that received prompt laser received fewer ranibizumab injections than needed [47].

The extension from 24 to 36 months of the READ study found that, in the ranibizumab group, the variation in BCVA from month 24 to month 36 was +3.1 letters (*p* = 0.009) and foveal thickness was thinner at month 36 than at month 24 (mean variation −70 microns, *p* = 0.006) [48]. The results of this study found that although long-term functional and anatomical outcomes with ranibizumab were very good, many patients required frequent injections to achieve such outcomes [48].

The long-term (three years) outcomes of the RISE and RIDE studies were published in 2013 [49]. RISE and RIDE were two phase III, multicenter clinical trials that were sham-controlled for two years. Patients were randomly assigned in a 1 : 1 regime to monthly sham injection or intravitreal ranibizumab (either 0.3 or 0.5 mg) during a follow-up period of two years. In the third year, sham patients, while still masked, were eligible to crossover to monthly 0.5 mg ranibizumab [49]. Functional outcomes at month 36 observed in the ranibizumab groups were in line with those previously reported in the two-year follow-up study [46]. The proportion of patients achieving a gaining ≥15 letters in BCVA was greater, in both RISE and RIDE, in the ranibizumab groups (independently of the dose) than in the sham group [49]. Patients who previously received sham when were changed to ranibizumab 0.5 showed lower BCVA improvements than those obtained by ranibizumab after their first year of treatment [49]. The incidence of serious adverse events potentially related to systemic VEGF inhibition was 19.7% and 16.8% in eyes treated with ranibizumab 0.3 mg and 0.5 mg, respectively [49].

The RESTORE extension evaluated the results of the protocol RESTORE after three years of follow-up. The RESTORE had a 12-month double-masked phase and a 24-month open-label extension [50]. 79% (240/303) of the patients who completed the first phase of the RESTORE were included in the extension, and 208 patients (86.7%) completed the extension study. In those patients previously treated with ranibizumab during the 12-month double-masked phase, BCVA improvement and CMT reduction were maintained. In the laser group, when ranibizumab intravitreal injections were allowed, there were a BCVA improvement (+6.0 letters) and a retinal thickness reduction (−142.7 *μ*m) at month 36. Cataract, with an incidence of 16.3%, was the most frequently reported adverse event. Eight patients died during the study; none were suspected to be related to the study drug/procedure [50].

The LUCIDATE study was a prospective, randomized, single-masked, and single-center clinical trial that compared the functional and anatomical effects of laser versus ranibizumab in DME patients [51]. Patients were randomly assigned in a 2 : 1 ratio to 3 monthly doses of ranibizumab and retreatment when needed vs. laser at baseline and repeated every 12 weeks (as needed) [51]. Mean BCVA improvement was +6.0 letters in the ranibizumab group vs. −0.9 letters in the laser group. Additionally, retinal sensitivity and electrophysiology function were improved with ranibizumab. Anatomic outcomes were better in the ranibizumab group than in the laser group. No safety issues were reported [51].

The 5-year results of the DRCR.net study evaluating ranibizumab plus prompt or deferred laser or triamcinolone plus prompt laser for diabetic macular edema suggested that, in eyes with DME, focal/grid laser treatment at the initiation of intravitreal ranibizumab treatment did not provide better results than deferring laser treatment for ≥24 weeks [52]. Additionally, deferring laser was also associated with an increase in the number of intravitreal injections of ranibizumab [52].

The REVEAL study was a 12-month, randomized, double-masked, multicenter, laser-controlled, phase III study conducted in the Asian population that compared the efficacy of three different therapeutic strategies: ranibizumab + sham laser, ranibizumab + active laser, or sham injection + active laser [53]. Ranibizumab alone or in combination with laser provided better functional and anatomic outcomes than laser alone. The most frequently reported ocular adverse event was conjunctival hemorrhage. There was no evidence of adverse events associated with systemic inhibition of VEGF [53].

The RELIGHT study evaluated the potential benefits of tailoring the ranibizumab treatment regime to the DME patient's needs [54]. Patients received initially a loading dose of 3 initial intravitreal ranibizumab injections (baseline and months 1 and 2). Based on individualized BCVA and CMT, patients received monthly intravitreal ranibizumab injections (months 3 to 5) and bimonthly between months 6 and 18 [54]. The results of this study suggested that functional outcomes obtained during the initial 6-month treatment regime were maintained during the bimonthly tailored treatment [54].

Similarly, the RETAIN study was designed to evaluate whether an incremental extension of intertreatment intervals (1 to 3 months) would be feasible [55]. In terms of functional outcomes, the incremental extension regimen did not provide worse results than a standard pro re nata (PRN) regime (monthly followed and treated according to signs of disease activity). However, the number of intravitreal injections was slightly greater with the incremental extension regimen [55].

The READ-3 study was a multicenter, prospective, and randomized study that compared the efficacy of intravitreal injections of ranibizumab 2.0 mg with ranibizumab 0.5 mg in eyes with DME [56]. The results of this study suggested that BCVA improvement was not superior with ranibizumab 2.0 mg than with ranibizumab 0.5 mg. The safety profile of both doses was similar [56].

The open-label extension of the RISE and RIDE protocols tried to answer the question of whether an intravitreal ranibizumab “less-than-monthly injection” regime would be as effective as monthly injection regime [57]. According to the results of this study, the functional and anatomic outcomes achieved with monthly ranibizumab might be maintained with a reduction in treatment frequency [57].

The efficacy and safety of ranibizumab and bevacizumab were compared directly in a randomized, double-masked, 36-week, and 3-period crossover study [58]. Patients received monthly intravitreal injections of bevacizumab (1.25 mg) or ranibizumab (0.3 mg). The results of this study found a slightly but statistically significant difference in BCVA improvement (difference of 1.3 letters in favor of ranibizumab; *p* = 0.039) and in the mean CMT reduction (difference of 48 *μ*m in favor of ranibizumab; *p* < 0.001) [58].

The question of whether early visual acuity response to ranibizumab in DME patients is associated with long-term outcomes was evaluated in a post hoc analysis of the DRCR.net Protocol I [59]. The results of this study suggested that a poor early response was associated with a poor long-term visual outcome. Additionally, this study found that 39.7% of the eyes underwent ranibizumab (with or without laser) did not adequately respond (BCVA improvement < 5 letters) at week 12 [59].

The two-year effectiveness of intravitreal ranibizumab in combination with a nutritional supplement rich in docosahexaenoic acid plus antioxidants was evaluated in a prospective, randomized, and single-blind controlled study [60]. At month 24, the combined therapy provided a statistically significant CMT reduction as compared with intravitreal ranibizumab alone. However, there were no significant differences in BCVA improvement between the two strategies [60].

The TREX-DME study was a multicenter, prospective, and randomized clinical trial designed for comparing monthly ranibizumab injections vs. an incremental extension algorithm [61]. The results of this study suggested that the incremental extension strategy did not provide worse functional or anatomic outcomes, while decreasing the number of injections administered [61].

The ROTATE trial was a prospective and open-label study that evaluates the efficacy of intravitreal of ranibizumab 0.3 mg in eyes with persistent DME after intravitreal bevacizumab treatment [62]. The results of this study showed that the mean BCVA was significantly improved (+6.5 letters) and CMT was significantly reduced (−116 *µ*m) under intravitreal ranibizumab treatment. On the negative side, systemic adverse events included two deaths, stroke, and myocardial infarction [62].

The RELATION study, a prospective, double-masked, multicenter phase IIIb trial, assessed the efficacy and safety of intravitreal ranibizumab 0.5 mg plus laser versus laser monotherapy in patients with DME [63]. As compared with laser monotherapy, ranibizumab + laser provided a significantly greater BCVA improvement (mean difference between groups, 4.2 letters; *p* = 0.001). However, there was no significant difference in CMT reduction between the two groups (*p* = 0.28) [63].

The REFINE was a phase III, 12-month, double-masked, multicenter, laser-controlled study conducted on Chinese patients with DME. Patients were randomly assigned (4.1) to receive intravitreal ranibizumab injections or laser [64]. The mean BCVA improvement with ranibizumab at month 12 (+7.9 letters) was statistically significantly greater (*p* < 0.001) than that observed with laser (+2.5 letters) [64].

Table 3 summarizes the main results of ranibizumab observed in different studies included in this review.

*(1) Safety*. As regards the safety profile, the incidence and characteristics of the adverse events were similar to that observed with bevacizumab. Systemic VEGF inhibition-related adverse events such as stroke and myocardial infarction have been described with the administration of ranibizumab [45–64]. The most commonly reported ocular adverse event among the different studies was conjunctival hemorrhage. Other ocular side effects included endophthalmitis, ocular hypertension, and retinal detachment [45–64].

Repeated intravitreal ranibizumab injections may increase the risk of ocular hypertension [65]. According to the results of the DRCR.net study, repeated intravitreal injections of ranibizumab were associated with a greater probability of sustained intraocular pressure (IOP) elevation than laser treatment (hazard ratio 2.9, *p* = 0.01) [65].

Regarding the potential negative effect on corneal endothelium of ranibizumab or bevacizumab, monthly intravitreal 0.5 ranibizumab or 1.25 mg bevacizumab during three months did not show any negative effect on corneal endothelium [66].

#### 3.1.4. Aflibercept

Aflibercept (EYLEA®; Bayer HealthCare, Berlin, Germany/Regeneron Pharmaceuticals Inc., Tarrytown, NY, USA) is a fusion protein (115 kDa) comprising the second Ig domain of human VEGFR1, the third Ig domain of human VEGFR2, and the Fc region of a human IgG1 [67–69].

The first high-quality scientific evidence published about the role of aflibercept in the management of DME were the VIVID in Europe [70] and the VISTA in the United States [71].

VIVID and VISTA were two similarly designed randomized phase III trials that compared the efficacy and safety of two doses of intravitreal regimes of aflibercept vs. laser for DME treatment [70, 71]. After a monthly loading dose of 5 injections, aflibercept 2 mg was administered every 4 (IA4W) or every 8 weeks (IA8W) [70, 71]. After 100 weeks of follow-up, the proportion of patients achieving a BCVA improvement of ≥15 letters in VIVID was 38.2%, 31.1%, and 12.1% for the IA4W, IA8W, and laser treatment regimes, respectively (*p* < 0.0001), and in VISTA was 38.3%, 33.1%, and 13.0% for the IA4W, IA8W, and laser treatment regimes, respectively (*p* < 0.0001). The pooled mean BCVA improvement from baseline to week 100 was 10.7 and 10.3 for IA4W and IA8W, respectively. There were no significant differences in terms of BCVA improvement between IA4W and IA8W regimes [70, 71].

Based on these results, the European Medicines Agency (EMA), in 2014, and the Food and Drug Administration (FDA), in 2015, approved the use of aflibercept for treating DME. FDA approved a dose of 2 mg per injection (5 monthly injections as loading dose plus bimonthly injections thereafter), while EMA adds (to the aforementioned) the option to establish an incremental extension of intertreatment intervals after the first year of treatment [6].

A post hoc analysis of the VIVID and VISTA trials compared the effect of intravitreal aflibercept on functional and anatomic outcomes in DME patients with and without prior anti-VEGF treatment [72]. Mean BCVA improvement at week 100 in those eyes that did not receive previous anti-VEGF treatment was 12.0, 11.3, and +2.1 letters for the IA4W, IA8W, and laser treatment regimes, respectively. In previously treated eyes, mean BCVA improvements at week 100 were +10.9, +10.8, and −0.8 letters. At week 100, mean CMT reductions in previously treated eyes were 180.1 *μ*m, 196.4 *μ*m, and 94.1 *μ*m for the IA4W, IA8W, and laser treatment regimes, respectively. In eyes without previous anti-VEGF treatment, at week 100, mean CMT reductions were 200.0 *μ*m, 186.7 *μ*m, and 76.9 *μ*m for the IA4W, IA8W, and laser treatment regimes, respectively [72]. Functional and anatomic improvements were statistically significant with both intravitreal aflibercept regimes as compared with laser. There were no statistically significant differences in both functional and anatomic outcomes between the two intravitreal aflibercept regimes [72].

The ENDURANCE extension study was a phase IV, open-label study conducted on patients who completed the VIVID and VISTA DME trials [73]. During the ENDURANCE study, both interval between patient visits and intravitreal aflibercept injections were tailored according to the patient's needs [73]. Sixty patients were enrolled in the ENDURANCE study. The BCVA improvements achieved during the VISTA were maintained and stable (<1.5 letters) over the 12-month follow-up.

Similar to BCVA, mean CMT remained relatively stable during the ENDURANCE study [73]. Regarding treatment needs, 42 (70%) received ≥ 1 intravitreal injection of aflibercept (mean 4.5 injections), without any significant impact of the treatment received during the VISTA [73].

The results of the 148-week analysis from the VISTA and VIVID studies confirmed the previous findings [74]. BCVA improvements achieved with both intravitreal aflibercept regimes at week 52 and week 100 were maintained at week 148. As regards the safety profile, the findings were consistent with the previous reports [70–73].

A post hoc analysis of Protocol T found that, for those eyes with a baseline visual acuity <69 letters, the BCVA improvement achieved with aflibercept was statistically greater than that obtained with bevacizumab and ranibizumab at 1 year. However, at 2 years, aflibercept was only superior, in terms of BCVA improvement, to bevacizumab [75]. Regarding retinal thickness, in eyes with baseline visual acuity <20/50, reduction observed at 1 year with bevacizumab was lower than with the other anti-VEGF drugs, but at 2 years the differences had diminished [75].

A secondary analysis of the Protocol T compared changes in diabetic retinopathy severity during aflibercept, bevacizumab, or ranibizumab treatment for DME [76]. At 1 year, in eyes with no proliferative diabetic retinopathy, a significantly greater proportion of patients treated with aflibercept or ranibizumab had improvement in diabetic retinopathy severity as compared with bevacizumab (*p* = 0.004 for aflibercept vs. bevacizumab and *p* = 0.01 for ranibizumab vs. bevacizumab), but there was no difference between aflibercept vs. ranibizumab (*p* = 0.51) [76]. However, at 2 years, no treatment group differences were identified in the proportion of patients who had diabetic retinopathy improvement. As regards the eyes with diabetic retinopathy, at 1 year a significantly greater proportion of patients treated with aflibercept had improvement in diabetic retinopathy severity as compared with bevacizumab (*p* < 0.001) or ranibizumab (*p* = 0.02), but not between ranibizumab and bevacizumab (*p* = 0.09) [76].

Unlike eyes with no proliferative diabetic retinopathy, these rates and treatment group differences seemed to be maintained at 2 years [76].

The 2-year outcomes of the ENDURANCE extension study found that the number of intravitreal aflibercept injections was substantially reduced in the fourth and fifth years of aflibercept dosing following initiation of therapy in the VISTA DME trial [77]. BCVA improvements achieved during the 3-year VISTA trial were maintained [77].

An additional post hoc analysis of the Protocol T evaluated the proportion of eyes with persistent DME after 24 weeks of treatment with aflibercept, bevacizumab, or ranibizumab. Persistent DME through 24 weeks was significantly less frequent with aflibercept than with the other treatments (*p* < 0.001 vs. bevacizumab and *p* = 0.05 vs. ranibizumab) and less frequent with ranibizumab than with bevacizumab (*p* < 0.001) [78]. Although the proportion of eyes with persistent DME was significantly lower with aflibercept, 31.6% of the eyes (60/190) did not adequately respond to this treatment [78].

An integrated post hoc subanalysis of the two phase II trials VISTA and VIVID assessed the effect of baseline factors on differences in BCVA improvement with intravitreal aflibercept injection vs. laser in DME patients [79]. According to the results of this study, BCVA improvement was significantly greater with aflibercept than with laser and was not influenced by any baseline factor [79].

The Protocol V was a prospective and randomized clinical trial that compared three different strategies for treating eyes with DME and good visual acuity (20/25 or better) [80]. Study eyes were randomly assigned in a 1 : 1 : 1 ratio to 2.0 mg of aflibercept, focal/grid laser photocoagulation, or observation. In the laser photocoagulation and observation groups, it was allowed to start with aflibercept if visual acuity met specific worsening criteria. At 2 years, there were no significant differences in the proportion of eyes with at least a 5-letter visual acuity decrease (aflibercept vs. laser, *p* = 0.79; aflibercept vs. observation, *p* = 0.79; and laser vs. observation, *p* = 0.79). In other words, in eyes with DME and good visual acuity, aflibercept or laser photocoagulation appeared to be no superior to observation [80].

The real-world functional and anatomic outcomes of intravitreal aflibercept in DME patients, either naïve or previously treated, were assessed in a prospective, observational, and multicenter cohort study conducted in France [81]. The APOLLON evaluated, as the primary outcome, the mean change in BCVA from baseline to month 12. The study included 147 patients (77 treatment-naïve and 70 previously treated) followed up for at least 12 months. The mean improvement in BCVA at month 12 was 7.8 ± 12.3 and 5.0 ± 11.3 letters in treatment-naïve and previously treated patients, respectively, *p* = 0.1541 (independent-sample Student's *t*-test) [81]. Intravitreal aflibercept significantly reduced CMT in both groups, without differences between them. The mean intravitreal injection administered during the study was 7.6 ± 2.5 in the treatment-naïve group and 7.6 ± 2.3 in the previously treated one, *p* = 1.000 (independent-sample Student's *t*-test) [81].

Table 4 summarizes the main results of ranibizumab observed in different studies included in this review.

*(1) Safety*. The incidence of either ocular or systemic side effects did not significantly differ from those reported for bevacizumab or ranibizumab [35, 36, 70–81].

Ziv-aflibercept (Zaltrap, Sanofi-Aventis US, LLC, Bridgewater, New Jersey, USA, and Regeneron Pharmaceuticals, Inc., Tarrytown, New York, USA), a recombinant fusion protein, has a mechanism that is similar in action to that of aflibercept and is available at a lower cost than the proprietary anti-vascular endothelial growth factor (VEGF) drug [82, 83].

The results of a 3-month prospective study, which included 17 eyes with DME, found that off-label use of intravitreal ziv-aflibercept improved visual acuity, without detectable ocular toxicity or systemic side effects in DME [84].

Additionally, the results of a retrospective study that evaluated the clinical outcomes and safety profile of ziv-aflibercept in eyes that received ≥10 intravitreal injections found that multiple intravitreal injections of ziv-aflibercept were associated with a significant improvement in both functional and anatomic outcomes, with a good safety profile [85].

#### 3.1.5. Conbercept

Conbercept is a recombinant soluble VEGF receptor decoy [86]. Its affinity for VEGF is 50 times that of bevacizumab and 30 times that of ranibizumab [87].

A retrospective study compared the efficacy of intravitreal conbercept either alone or in combination with laser [88]. The results of this study suggested that both treatment strategies significantly improved BCVA and reduced CMT, without differences between them (*p* = 0.164 and *p* = 0.149 for the BCVA and CMT, respectively) [88]. Nevertheless, the number of intravitreal injections of conbercept was significantly lower with the combined therapy (3.3 ± 1.2 per eye) than with conbercept alone (5.6 ± 0.8 per eye), *p* < 0.001 [88].

The efficacy of intravitreal conbercept and ranibizumab for treating DME was evaluated in a 12-month, retrospective, and real-life study [89]. Patients received intravitreal conbercept injections or intravitreal ranibizumab injections, once a month for 3 months followed by as-needed therapy. At month 12, BCVA improvement was 9.3 ± 5.2 and 8.9 ± 4.4 in the conbercept and ranibizumab groups, respectively, *p* < 0.001 each (with no significant differences between groups, *p* = 0.756). At month 12, the mean CMT reduction was 138.4 ± 97.7 *μ*m in the conbercept group and 145.2 ± 72.5 *μ*m in the ranibizumab group, *p* < 0.001 each (with no significant differences between groups, *p* = 0.748) [89].

The efficacy and safety of intravitreal conbercept for the treatment of DME were evaluated in a retrospective study. The BCVA improvement at months 1 and 3 was sadistically significant; however, such improvement started to decrease at month 6 [90]. Notably, 32.6% of the eyes treated with intravitreal conbercept were not sensitive to it within half a year. CMT reduction was basically maintained at month 12 [90].

Moreover, a meta-analysis that compared the efficacy of conbercept and ranibizumab for the treatment of DME reported that intravitreal conbercept was significantly superior to ranibizumab in terms of CMT reduction, but no statistically significant difference with regard to visual improvement [91].

Additionally, it appears that conbercept was able to significantly improve the BCVA independently of the baseline visual acuity, although for worse baseline visual acuity (20/50 or worse), BCVA improvement was more prominent than that of better baseline visual acuity (20/32 to 20/40) subgroup [92].

The results of a meta-analysis, which included 588 patients, compared the effect and safety of conbercept and ranibizumab in the treatment of DME suggested that intravitreal injections of conbercept were superior to ranibizumab in both reducing CRT and improving BCVA [93]. Regarding safety, the pooled results showed that there was no significant difference in the risk of intraocular pressure increase (or conjunctival hemorrhage between two groups [93].vd

*(1) Safety*. The most frequently reported ocular adverse event was conjunctival hemorrhage [88–93]. The incidence and type of adverse events did not significantly differ from those previously reported for other anti-VEGF therapies (Sections 3.1.2to 3.1.4).

### 3.2. Steroids

Since there is increasing evidence about the role of inflammation on the pathophysiology of DME, corticosteroids have taken an active role in its treatment [8–11]. Corticosteroid therapy is able to inhibit many of the processes known to be involved in the progression of DME, through anti-inflammatory properties [94] and VEGF inhibition [95]. Corticosteroids stabilize retinal capillaries and tend to reduce their permeability decreasing the leakage of plasma proteins into the interstitial tissue compartment [8, 9, 96].

Although a single-dose preparation of preservative-free triamcinolone acetonide (Triesence®; Alcon Laboratories, Inc., Fort Worth, TX, USA) has been approved by the FDA to enhance visualization of the vitreous during pars plana vitrectomy and to treat some posterior segment inflammatory diseases [97], it has not been approved for the treatment of DME. That is why triamcinolone acetonide would not be analyzed in this review.

#### 3.2.1. Dexamethasone Sustained-Release Implants

Dexamethasone intravitreal (DEX) implant (0.7 mg) (Ozurdex, Allergan, Inc., Irvine, CA, USA) consists of micronized dexamethasone in a biodegradable copolymer of polylactic-co-glycolic acid which slowly releases steroids into the vitreous over a period of about 6 months [98, 99]. In 2014, based on the results of the MEAD study [100], the FDA and most European countries approved Ozurdex for the treatment of DME.

The first prospective, randomized, and controlled trial evaluating the efficacy and safety of an intravitreal DEX in eyes with DME was published in 2010 [101]. Eyes with persistent DME (≥90 days of duration) were randomly assigned to receive a DEX implant (700 *µ*g or 350 *µ*g) or observation. At month 3, the proportion of eyes achieving a BCVA improvement of ≥10 letters was 33.3%, 21.1%, and 12.3% in the 700 *µ*g DEX, 350 *µ*g DEX, and controls, respectively (700 *µ*g DEX vs. controls, *p* = 0.007) [101].

Additionally, better anatomic outcomes were observed with the 700 *µ*g DEX than in the control group (*p* = 0.03). However, at month 6, there were no significant differences between groups [101]. According to the results of this clinical trial, 700 *µ*g DEX was an effective option for treating persistent DME, but its effects seemed to be time-limited [101].

The CHAMPLAIN was a prospective, multicenter, open-label, and 26-week study that evaluated the efficacy and safety of a 700 *µ*g DEX for the treatment of DME in vitrectomized eyes [102]. At week 26, 700 *µ*g DEX significantly reduced the mean CMT (*p* = 0.004) and significantly improved the mean BCVA (*p* = 0.046) as compared to baseline. At week 8, the proportion of eyes that achieved a BCVA of ≥10 letters were 30.4% [102]. The most commonly reported ocular adverse events were conjunctival hemorrhage, conjunctival hyperemia, elevation of IOP, and eye pain [102].

The PLACID study was a multicenter, prospective, randomized, controlled, double-masked, parallel-group, and 12-month clinical trial that compared the 0.7 mg DEX implant (Ozurdex^®^) in combination with laser therapy vs. laser alone for the treatment of diffuse DME [103]. A total of 253 DME patients were randomly assigned to 0.7 mg DEX + laser (at month 1) or sham implant + laser. Patients could receive up to 3 additional laser treatments and 1 additional DEX implant or sham treatment as needed [103]. The proportion of eyes with a BCVA improvement of ≥10 letters was significantly greater at months 1 and 9 in the combination group than in the laser group (*p* < 0.001 and *p* = 0.007, respectively), although at month 12 such a difference was not significant [103]. Additionally, the area of vascular leakage was significantly decreased in the combination group as compared with the laser group (*p* = 0.041). Regarding safety, the incidence of elevation of IOP was significantly greater in the combination therapy group than in the laser group, but no eyes in the combination group required glaucoma surgery. Cataract-related side effects were more frequent in the combination group (22.2%) than in the laser group (9.5%), although there was no difference in the number of cataract surgeries between groups (4 eyes in the combination group and 5 eyes in the laser alone group) [103].

One of the most important studies assessing the effect of DEX treatment in DME patients was the MEAD [100]. The MEAD was two prospective, multicenter, randomized, masked, sham-controlled, three-year, and phase III clinical trials designed to evaluate the efficacy and safety of DEX implant 0.7 and 0.35 mg in patients with DME [100]. At baseline, study patients were randomly assigned (1 : 1 : 1) to receive DEX implant 0.7 mg, DEX implant 0.35 mg, or a sham procedure. The proportion of patients achieving a BCVA improvement of ≥15 letters at year 3 (or at the last study visit) was significantly greater with both DEX implant 0.7 mg (22.2%, *p* < 0.001 vs. sham) and DEX implant 0.35 mg (18.4%, *p* = 0.018 vs. sham). Interestingly, the treatment effect was really fast; in fact, significant differences in the proportion of eyes gaining ≥15 letters were observed as early as day 21. The mean CMT reduction was significantly greater with 0.7 mg DEX (−111.6 ± 134.1 *μ*m) and 0.35 mg DEX (−107.9 ± 135.8 *μ*m) than with sham (−41.9 ± 116.0 *μ*m), *p* < 0.001 each [100].

Among phakic eyes at baseline, the incidence of cataract-related side effects was 67.9%, 64.1%, and 20.4% in the 0.7 mg DEX, 0.35 mg DEX, and sham, respectively. The rate of cataract surgery was 59.2%, 52.3%, and 7.2% in the 0.7 mg DEX, 0.35 mg DEX, and sham, respectively. An increase in IOP was observed in 27.7%, 24.8%, and 3.7% of the eyes underwent 0.7 mg DEX, 0.35 mg DEX, and sham, respectively. Five (1.4%) eyes in the 0.7 DEX, 3 (0.9) in the 0.35 DEX, and 1 (0.3%) sham required a glaucoma procedure (trabeculoplasty, iridotomy, iridectomy, or trabeculectomy) [100].

The BEVORDEX study was a phase II, prospective, multicenter, randomized, single-masked clinical trial that compared the 0.7 DEX implant Ozurdex vs. bevacizumab in patients with DME [104]. This study enrolled 88 eyes from 61 patients who were randomized to receive DEX (46 eyes) every 16 weeks or bevacizumab (42 eyes) every 4 weeks, both PRN. At month 12, the proportion of eyes having a BCVA improvement of ≥10 letters was 41 % (19/46) and 40% (17/42) in the DEX and bevacizumab groups, respectively (*p* = 0.83). The mean CMT reduction was significantly greater with DEX (187 *μ*m) than with bevacizumab (122 *μ*m), *p* = 0.015. The mean number of intravitreal injections with DEX (2.7) was significantly lower than with bevacizumab (8.6) [104].

The eyes included in the BEVORDEX study continued in the trial for another year on the same treatment allocation, and 68 (77%) of the 88 enrolled eyes completed the 24-month trial [105]. The results of the BEVORDEX study at 2 years found that 43% (20/46) DEX and 45% (19/42) bevacizumab-treated eyes achieved a BCVA improvement of ≥ 10 letters (*p* = 0.99) (105). At month 24, there were significant differences between groups in the mean CMT reduction. Although, during the second year, the mean number of intravitreal injections with bevacizumab (4.8 ± 5.1) was greater than that of DEX (2.2 ± 1.2), the difference was less pronounced than during the first year [104, 105].

The efficacy and safety of DEX implant in DME eyes that did not adequately respond to three monthly intravitreal injections of anti-VEGF were evaluated in a prospective clinical trial [106]. The results of this study suggested that DEX significantly improved BCVA at month 2 (*p* = 0.0381) and significantly reduced CMT at months 1, 2, and 3 (*p* = 0.0343, *p* = 0.0288, and *p* = 0.0370, respectively). In the negative side, as compared with baseline, the IOP was significantly greater at months 1, 2, and 3 (*p* = 0.0003, *p* = 0.0003, and *p* = 0.0048, respectively) [106].

The results of the BEVORDEX 12-month study [104] were confirmed by a single-center, randomized, and subject-masked study conducted on eyes with persistent DME [107]. The results of this study found no differences in the mean change in BCVA between DEX (+5.8 ± 7.6 letters) and bevacizumab (+5.6 ± 6.1) (*p* = 0.785). Nevertheless, the mean change in CMT was significantly greater with DEX (−122 ± 120 *μ*m) than with bevacizumab (−13 ± 105) (*p* < 0.001). Similarly, in the BEVORDEX 12-month study (171), the number of injections was significantly greater with bevacizumab (7.0 ± 0.2) than with DEX (2.7 ± 0.5) (*p* < 0.001) [107].

A single-masked, randomized controlled study determined whether combined therapy with DEX + intravitreal bevacizumab (1.25 mg) provides better outcomes than bevacizumab monotherapy in DME eyes [104]. At month 12, BCVA improvement was significant and equivalent in both groups (+5.4 and + 4.9 in the combined and bevacizumab monotherapy groups, respectively, *p* = 0.75). Nevertheless, the central subfield thickness reduction was significantly greater with the combined therapy than with bevacizumab alone (mean difference, 69 *μ*m, 95% confidence interval = 9–129; *p* = 0.03) [108].

Data collected from the MEAD trials were pooled [109]. This post hoc analysis aimed at comparing the long-term effects of DEX (either 0.7 mg or 0.35 mg) on the anatomic outcomes. Patients were randomized (1 : 1 : 1) to intravitreal DEX implant 0.7 mg, DEX implant 0.35 mg, or a sham procedure in the study eye. Of the 1,048 randomized patients of the intend-to-treat population, 607 (57.9%) patients completed all visits. At the end of the study follow-up, the mean CMT reduction was significantly greater with both 0.7 DEX (117 *μ*m) and 0.35 mg DEX (127.8 *μ*m) than with the sham (62.1 *μ*m), (both *p* < 0.001 vs. sham) [109].

The UDBASA was a multicenter, prospective, and randomized study, conducted on patients with DME, designed to evaluate a single administration vs. a PRN administration of a DEX [110]. Patients were randomly assigned to two groups: In Group I, patients were treated according to the MEAD protocol (only one DEX during the 6 months of follow-up) [100]; in Group II, patients were treated on as‐needed basis (once received the first DEX, patients visited every month and based on BCVA and CMT receive a customized PRN treatment regime) [110]. As compared to baseline, BCVA significantly improved, in both groups, at months 1 and 3, and started to decline in Group I at month 6. Although a difference of 0.11 logMAR in BCVA between groups was observed (in favor of the PRN regime), it was not statistically significant. A statistically significant CMT reduction in both groups was observed up to month 2, but at that time Group I had begun to revert to pretreatment level. CMT reduction from baseline at months 4 and 5 was statistically significant in favor of the PRN regime (*p* < 0.05). The mean number of DEX in Group II was 1.6 vs. 1 in Group I [110]. The proportion of patients with an increased IOP requiring glaucoma medical therapy was 14% and 30% in Groups I and II, respectively (*p* = 0.13). The need of cataract surgery was similar in both groups (48% and 40% in Groups I and II, respectively, *p* = 0.26 [110].

A phase II prospective, randomized, and multicenter clinical trial compared, in patients with persistent DME, the effect of two treatment strategies: continued ranibizumab alone vs. continued ranibizumab plus DEX [111]. DME pseudophakic eyes with a BCVA score of between 24 and 78 letters and previously treated with anti-VEGF therapy (at least 3 anti-VEGF injections) were included in the study. There was a run-in period, where the patients received treatment with 3 intravitreal injections of ranibizumab. Those eyes that met the inclusion/exclusion criteria after the 12-week run-in period were randomized (1 : 1) to receive either intravitreal ranibizumab + sham implant or intravitreal ranibizumab + DEX. BCVA improvement was similar in both groups (mean difference, 0.5 letters, *p* = 0.73). The mean CMT reduction was significantly greater in those eyes treated with ranibizumab + DEX than in those who receive ranibizumab alone (mean difference, 52 *μ*m; *p* < 0.001). The incidence of either increased IOP or initiation of glaucoma medical therapy was significantly higher in the ranibizumab + DEX (29%) than in the ranibizumab alone treated eyes (0%), *p* < 0.001 [111].

Table 5 summarizes the main results of the DEX implant.

Besides the good efficacy and safety profile of DEX reported in the clinical trials [100–111], the efficacy and safety of Ozurdex® for the treatment of DME have been recently evaluated in clinical and real-life studies [113–120]. In summary, the results of these studies clearly indicated that Ozurdex® significantly improved the functional (visual acuity) and anatomic (retinal thickness) outcomes, not only in the midterm [113, 114, 119, 120] but also in the long term [115–118], in both naïve and previously treated DME patients, but naïve eyes consistently fared better [113–118, 120].

Malclès et al. [116], in a retrospective and bicentric study, evaluated the efficacy and safety of DEX in DME patients in real-life practice over a period of 3 years. The results of this study found a significant improvement in BCVA (9.5 letters at month 36, *p* = 0.023), with 25.4% of eyes achieving at least a 15-letter improvement at month 36. Additionally, there was a significant decrease in CMT at month 36.

A retrospective and multicenter study assessed the efficacy and safety of repeated DEX, over a 24-month follow-up period, in DME eyes, either naïve or refractory to anti-VEGF, in a real setting [118]. The results of this study showed that although both, naïve and refractory eyes, improved significantly in vision after 24 months (*p* < 0.001), BCVA improvement was significantly greater in the naïve eyes that in the refractory ones (*p* < 0.01.) A statically significant CMT reduction was observed in both groups [118].

When treating DME patients, one important question is to know when to change a treatment strategy and what treatment to choose. Despite that the anti-VEGF therapy has been chosen as first-line therapy [6], many eyes do not adequately respond to them. A post hoc analysis of the DRCR.net Protocol I revealed that 40% of eyes achieved a BCVA improvement <5 letters at week 12 [59]. Additionally, eyes with a poor response to ranibizumab (those gaining <5 letters after three intravitreal ranibizumab injections administered monthly) usually do not improve further with continuing in ranibizumab treatment [59].

Moreover, extending the dose to 24 weeks did not provide better functional or anatomic outcomes [78].

However, the question of whether patients who do not adequately respond to anti-VEGF could benefit from an early change to another therapy has not been fully elucidated.

A retrospective, multicenter, and case-control study, conducted in a real setting, compared the effect of continuing with an anti-VEGF therapy or switching to a DEX in eyes with refractory DME after three initial anti-VEGF injections [121]. One hundred and ten (72 eyes in the anti-VEGF and 38 in the DEX groups) were included in the study. The mean change in BCVA was significantly greater in the DEX (+6.1 ± 10.6 letters) than in the anti-VEGF (+0.4 ± 10.8 letters) group, *p* = 0.004. The mean CMT reduction was significantly greater in the DEX (− 92.8 ± 173.6 *µ*m) than in the anti-VEGF (+18.3 ± 145.9 *µ*m) group, *p* < 0.001. At month 12, the probability of achieving a BCVA improvement of ≥10 letters was significantly greater in the DEX than in the anti-VEGF group (odds ratio, 3.71; 95% confidence interval, 1.19–11.61; *p* = 0.024) [121].

The effect of early (DME eyes receiving 3 or fewer anti-VEGF injections before switch) vs. late switch (DME eyes receiving 6 or more anti-VEGF injections before switch) on BCVA and CMT was compared in a retrospective study [122]. As compared to baseline, BCVA significantly improved in the early-switch group at month 24 (*p* = 0.043) but did not in the late-switch group (*p* = 0.8602). The CMT was significantly reduced in both early- and late-switch groups (*p* = 0.0002 and *p* = 0.0038, respectively). Nevertheless, the proportion of eyes obtaining a CMT reduction ≥10% was significantly greater in the early-switch group than in the late-switch one (71.0% vs. 47.4%, *p* = 0.0498). There was no difference in the incidence of IOP increase between both groups [122].

These results were partially confirmed by a retrospective study, which found that, at month 6, the change in central retinal thickness was significantly better in the early-switch group than in the late-switch group [123].

Finally, the results of a retrospective study published recently suggested that, in DME patients who did not adequately respond to 3 monthly intravitreal anti-VEGF injections, switching to dexamethasone implant provided better functional outcomes than those that received >3 anti-VEGF injections [124].

*(1) DEX Implant in Cataract Surgery*. Cataract surgery is a common and safe procedure but can be associated with vision-threatening complications in the diabetic population, such as diabetic macular edema, postoperative macular edema, diabetic retinopathy progression, and posterior capsular opacification [125, 126].

Different hypotheses about the mechanisms involved in the pathogenesis of cataract in diabetic patients have been proposed, including polyol pathway, osmotic and oxidative stress, or autoimmunity [127]. Besides preoperative counselling, which is crucial for diabetic patients, other aspects such as glycemic control, evidence of ocular inflammation, history of preexisting proliferative diabetic retinopathy, and/or macular edema should be taken into consideration before cataract surgery in diabetic patients [125–127].

Since preexisting DME can increase the risk of macular edema progression by 20%–50%, an appropriate therapeutic management of DME is recommended perioperatively [128]. Surgical inflammation associated with cataract surgery may be responsible for poor functional outcomes in DME patients [125–127]. Moreover, diabetic patients have a substantial risk of developing DME after cataract surgery and particularly in the 3- to 6-month postoperative period [125]. Therefore, the perioperative administration of a DEX implant in diabetic patients undergoing cataract surgery might be beneficial.

We have evidence suggesting that, in diabetic patients, the intraoperative use of a DEX implant in combination with phacoemulsification and IOL implantation could provide good functional and anatomic outcomes [129–131].

A prospective study, conducted on 19 eyes of patients with type 2 diabetes mellitus with DME, who underwent cataract surgery, found that intraoperative DEX implant effectively prevented DME worsening after phacoemulsification [129]. Similarly, Furino et al. [130] reported that intraoperative intravitreal DEX provided good functional and anatomic clinical outcomes in DME patients who underwent cataract surgery and these positive effects last for at least 3 months. Moreover, the results of a prospective, single-arm, and open-label study suggested that prophylactic use of intraoperative DEX resulted in excellent anatomic outcomes in DME undergoing cataract surgery [131].

A retrospective and comparative cohort study published recently compared anatomical and functional outcomes of combined phacoemulsification and dexamethasone intravitreal implant with standard phacoemulsification in patients with nonproliferative diabetic retinopathy, ME, and cataract [132]. The results of this study found that, in those patients who underwent combined phacoemulsification and DEX implant, there was a significant and maintained increase in BCVA and a significant decrease in central subfoveal thickness throughout the study. The IOP significantly increased during the follow-up in the combined phacoemulsification and DEX implant group, although it remained within the normal range. According to the results of this study, in diabetic patients with DME, combined treatment with phacoemulsification and DEX implant provided better functional and anatomic outcomes than standard phacoemulsification [132].

#### 3.2.2. Fluocinolone Sustained-Release Implants

The efficacy and safety of two intravitreal fluocinolone sustained-release (IFSR) devices 0.2 *μ*g/day (low-dose) or 0.5 *μ*g/day (high-dose) were assessed in DME patients. Two parallel, prospective, randomized, sham injection-controlled, double-masked, multicenter clinical trials (FAME trials) were conducted on patients with persistent DME who received at least 1 macular laser treatment. Study subjects were randomized (2.2 : 1) to low-dose implant, high-dose implant, or sham implant, respectively [112]. At month 24, the proportion of patients achieving a BCVA improvement of ≥15 letters was significantly greater in both low-and high-dose groups than in the sham group (*p* = 0.002 each). Mean BCVA improvement was 4.4, 5.4, and 1.7 in the low-dose, high-dose, and sham groups, respectively (low-dose vs. sham, *p* = 0.02, and high-dose vs. sham *p* = 0.016). As compared with sham, CMT reduction was significantly greater in both low and high dose at all the time points measured. The need of glaucoma surgery was 3.7%, 7.6%, and 0.5% in the low-dose, high-dose, and sham groups, respectively [112] (Table 5).

In a subgroup analysis of the FAME trials, the efficacy and safety of an IFSR 0.2 *μ*g/day or sham in eyes with chronic (duration of diagnosis, ≥3 years) and nonchronic (duration of diagnosis, <3 years) DME were assessed [133]. At month 36, proportion of patients gaining ≥15 letters in BCVA was significantly greater in chronic DME patients (IFSR 0.2 *μ*g/day, 34.0% vs. sham, 13.4%; *p* < 0.001), but not in patients with nonchronic DME (IFSR 0.2 *μ*g/day, 22.3% vs. sham, 27.8%; *p* = 0.275). The greater functional response observed in the chronic DME eyes was not associated with baseline ocular characteristics, changes in anatomic features, or differences in retreatment or ancillary therapies. Duration of DME did not influence the incidence of adverse events [133].

A post hoc analysis of the FAME trials evaluated the treatment outcomes in phakic eyes who received IFSR 0.2 *μ*g/day implant [134]. At month 36, the proportion of eyes achieving a BCVA improvement of ≥15 letters was slightly higher in the eyes that had cataract surgery after (35.1%) than in the eyes that had cataract surgery before (29.3%). Additionally, as compared with nonchronic DME (27.5%), a greater proportion of eyes with chronic DME (42.3%) achieved a BCVA improvement of ≥15 letters [134].

Elevated IOP was more common in IFSR than in sham control-treated patients. There was no evidence that either glaucoma surgery, laser, or topical medication significantly impacted visual outcomes. None of the previously treated eyes with steroids that receive treatment with an IFSR 0.2 *μ*g/day implant required IOP-lowering surgery [135].

The long-term efficacy and safety of an IFSR were assessed in a prospective, randomized, evaluator-masked, controlled, and multicenter study. Study patients were randomized (2 : 1) to receive either 0.59 mg IFSR or standard of care (additional laser or observation) [136]. At 2 years, the proportion of eyes achieving a BCVA improvement ≥3 lines were 31.8% and 9.3% in the IFSR and standard-of-care groups, respectively, *p* = 0.0016. However, at 3 years, such difference was not statistically significant (*p* = 0.1566). The proportion of eyes with no evidence of retinal thickening was significantly higher in the standard-of-care group than in the IFSR at month 6, and years 1 and 2 (*p* < 0.0001, *p* < 0.0001, and *p* = 0.016, respectively), but not at year 3 (*p* = 0.861). An IOP ≥30 mmHg (at any time point visit) was observed in 61.4% and 5.8% of the IFSR and standard-of-care groups, respectively. 33.8% of the eyes treated with IFSR required surgery for ocular hypertension by 4 years [136].

The approved dose for the fluocinolone acetonide implant (Iluvien^®^; Alimera Sciences, Inc., Alpharetta, GA, USA) was 0.19 mg. In theory, it is delivered over 36 months at a rate of 0.2 *μ*g/day [137]. However, pharmacokinetic studies reported that IFSR provides sustained delivery for approximately one year [138].

To date, there have been numerous papers looking at the real-world efficacy and safety profile of the IFSR implant at years 1 and 2 and data are emerging for 3 years. The results of these studies are in the same general direction, indicating that, on average, IFSR implant significantly improved BCVA and reduced CMT in DME patients [139–148].

The ILUVIEN Implant for chronic DiabEtic MAcuLar edema (Retro-IDEAL) was a retrospective study designed for assessing the efficacy and safety of an IFSR (0.19 mg) in patients with chronic DME in Germany [143]. The results at month 30 found that, from baseline, BCVA significantly improved (*p* < 0.05). Additionally, CMT was significantly reduced at year 3 (*p* < 0.001) [144].

### 3.3. Emerging Therapies

#### 3.3.1. Designed Ankyrin Repeat Protein (DARPin)

Designed ankyrin repeat protein (DARPin), which derives from natural ankyrin repeats, is a small, single-domain protein that can selectively bind to a target protein with high affinity and specificity [149, 150].

Besides their high selectivity and affinity, DARPin molecules also display remarkable stability that confer some advantages over currently available antibodies or antibody fragments as potential therapeutics. In addition, DARPin molecules can be specifically designed to modulate local or systemic pharmacokinetics [150, 151].

Abicipar pegol (AGN-150998, MP0112, abicipar; Allergan plc/Molecular Partners) is an antagonist of VEGF-A characterized by small size, high potency, and long intravitreal half-life [15, 152]. A phase I/II, open-label, multicenter dose-escalation trial, evaluating the safety and bioactivity of abicipar pegol in DME patients, found that there were prolonged edema reduction and improvement in vision [153]. Similarly, the results from a phase II study showed that abicipar pegol, injected every 8 or 12 weeks in patients affected by DME, offered the functional and anatomical effects with less frequent injections compared with ranibizumab over a 28-week period [154].

#### 3.3.2. Brolucizumab

Brolucizumab (Beovu®) is a low-molecular-weight, single-chain antibody fragment anti-VEGF developed by Novartis (Basel, Switzerland) for the treatment of neovascular age-related macular degeneration, DME, and macular edema secondary to retinal vein occlusion [155].

A 6 mg dose of brolucizumab delivers a molar dose which is about 11 and 22 times higher than aflibercept 2 mg and ranibizumab 0.5 mg, respectively.

This drug has been already compared to aflibercept in patients with neovascular age-related macular degeneration [156, 157]. The HAWK and HARRIER, two similarly designed phase III clinical trials conducted on patients with neovascular age-related macular degeneration, showed that brolucizumab was not inferior to aflibercept in visual function at week 48, although anatomic outcomes favored brolucizumab over aflibercept [157]. Regarding safety, it should be mentioned that intraocular inflammation was identified in 50 (4.6%) of the brolucizumab-treated patients. Of those, 36 subjects (3.3%) had concomitant retinal vasculitis. Of the 36 subjects with intraocular inflammation and vasculitis, 23 subjects (2.1%) had concomitant vascular occlusion [157]. The American Society of Retina Specialists (ASRS) conducted a postapproval analysis of brolucizumab-associated retinal vasculitis after cases [158]. Retinal vasculitis was reported in 26 eyes of 25 patients after treatment with brolucizumab (of which 85% were designated as occlusive). Twelve eyes (46%) had a greater than 3-line decrease in VA at the final follow-up, and 12 eyes (46%) had a final VA of 20/200 or worse [158]. Additionally, a retrospective case series found that retinal vasculitis and intraocular inflammation after intravitreal injection of brolucizumab were characterized by variable occlusion of large or small retinal arteries, or both, and perivenular abnormalities [159].

However, up to now, there are no published data in DME patients. A 2-year, randomized, double-masked, multicenter, active controlled study is ongoing to compare the efficacy and safety of brolucizumab 3 mg and brolucizumab 6 mg vs. aflibercept 2 mg. The estimated study completion date will be October 2021, and its results will be available in the next few years [160].

Additionally, the ongoing prospective, randomized, phase III clinical study in DME, KITE, aims to confirm the noninferiority of brolucizumab 6 mg compared to aflibercept 2 mg on a functional and morphological level as well as durability effect over 2 years [161]. However, its results have not been published yet.

#### 3.3.3. Angiopoietin Combination Drugs

Angiopoietins are a family of growth factors that bind to endothelial receptor tyrosine kinases [162]. Angiopoietin-2 is considered a key factor in DME pathogenesis (see Figure 1) [9, 152].

As far as we know, there are some ongoing clinical trials targeting angiopoietins in DME patients [11, 152]. The BOULEVARD trial was a prospective, randomized, and multicenter clinical trial that compared the efficacy and safety and efficacy of antibody targeting angiopoietin-2 and VEGF-A vs. ranibizumab in patients with DME [163]. The angiopoietin-2 inhibitor demonstrated statistically superior VA gains than ranibizumab at week 24 in treatment-naïve patients [163].

## 4. Conclusions

Many different options are currently available for treating DME. Although laser photocoagulation was considered the gold standard treatment for DME for several years, it is not longer.

Nowadays, intravitreal corticosteroids and anti-VEGF have become first line for treating DME. The anti-VEGF of choice might depend on baseline BCVA. While aflibercept may be the drug of choice in patients with low baseline BCVA (approximately 20/50 or worse); the three anti-VEGFs (bevacizumab, ranibizumab, and aflibercept) provide similar functional outcomes in patients with higher baseline BCVA (approximately 20/32 to 20/40).

Since the identification of the role of inflammation, corticosteroids have taken an active role in the treatment of DME. Intravitreal corticosteroids represent a valuable option for treating DME. However, they are usually seen as a second choice, particularly in those eyes that have an insufficient response to anti-VEGF. Emerging evidence suggests that, in eyes that did not adequately respond to 3 anti-VEGF injections, switching to a DEX implant provides better functional outcomes than in those who received >3 anti-VEGF injections. This finding brings to the table the convenience of not extending anti-VEGF further in those eyes that exhibited an insufficient therapeutic response after three doses.

Since there seems to be a relationship between anti-VEGF and vascular disorders, especially in elderly people, intravitreal corticosteroids would be the treatment of choice in patients at risk of suffering cardiovascular and/or cerebrovascular events. Another group of patients who could potentially benefit from corticosteroids as first-line therapy are those unwilling to follow the anti-VEGF treatment regime (monthly injections and/or monthly visits) during the first 6 months. At the time of choosing a corticosteroid, dexamethasone should be used first, reserving the use of fluocinolone for those DME cases that did not adequately respond to other treatments. Intravitreal triamcinolone is associated with a greater IOP increase and higher incidence of cataract, and its use should be reserved in those patients who cannot obtain the approved agents for this indication.

DARPins against VEGF have been demonstrated to inhibit specifically angiogenesis in both in vitro and in vivo studies. These treatments have shown to be as effective as anti-VEGF, but with fewer doses, which may reduce the risk of serious adverse events, for example, endophthalmitis.

New therapies will probably come in the next years for treating DME. Also, novel drug delivery systems using nanotechnology, sustained-release delivery implants, and stem cell therapy are on the horizon.

## Figures and Tables

**Table 1:** Overview of the functional and anatomic results of bevacizumab.

Study	Ref.	Duration (w)	Regimen	*N* (eyes)	BCVA (ETDRS letters)		CRT (*µ*m)	
					Baseline	Change^a^	Baseline	Change
DRCR.net^a^	[29]	12	Laser	19	64 (50 to 70)	−1 (−6 to 5)	441 (354 to 512)	−40 (−146 to 85)
			Beva I	22	65 (60 to 70)	5 (1 to 12)^*∗*^	397 (320 to 358)	−56 (−120 to −6)
			Beva II	24	63 (57 to 71)	7 (4 to 11)^*∗*^	446 (342 to 543)	−47 (−125 to −16)
			Beva III	22	64 (52 to 68)	4 (−3 to 7)	406 (353 to 520)	−5 (−41 to 53)
			Beva IV	22	66 (57 to 72)	0 (−5 to 8)	389 (308 to 452)	−40 (−103 to 33)

BOLT^b^	[30]	52	Beva 1.25 mg	42	55.7 (9.7)	8 (1 to 10)^*∗∗*^	507 (145)	−130 (122)
			Laser	38	54.6 (8.6)	−0.5 (−15 to 5)	481 (121)	−68 (171)
	[31]	104	Beva 1.25 mg	42	55.7 (9.7)	8.6^*∗∗*^	507 (145)	−146
			Laser	38	54.6 (8.6)	0.5	481 (121)	−118

Nepomuceno et al.^c^	[32]	48	Beva 1.5 mg	32	0.60 (0.05)	0.36 (0.05)	451.7 (22.3)	−122.0 (20.9)
			Rani 0.5 mg	28	0.63 (0.06)	0.34 (0.04)	421.9 (23.1)	−141.0 (18.6)

Kriechbaum et al.^c^	[33]	52	Beva 2.5 mg	15	0.30 (0.19 to 0.42)	0.18 (0.06 to 0.3)	505 (438 to 572)	351 (258 to 445)
			Triam 8 mg	15	0.32 (0.2 to 0.43)	0.36 (0.19 to 0.52)	490 (433 to 547)	296 (224 to 368)

Sonoda et al.^c^	[34]	12	Beva 1.25 mg	26	0.48 (0.32)	0.40 (0.25)	495.7 (195.3)	449.7 (212.2)
			Triam 4 mg	25	0.40 (0.25)	0.31 (0.23)	503.9 (171.4)	389.4 (209.4)

*Note*. ^a^Data are expressed in median (interquartile range). ^b^Data are expressed in mean (standard deviation). ^c^Mean (standard deviation) best-corrected visual acuity (logMAR) at baseline and at the last follow-up visit. ^*∗*^*p* < 0.01 vs. laser. ^*∗∗*^*p* < 0.001 vs. laser. w: weeks; BCVA: best-corrected visual acuity; EDTRS: Early Treatment of Diabetic Retinopathy Study; CRT: central retina thickness; DRCR.net: Diabetic Retinopathy Clinical Research Network; Beva I: intravitreal injection of 1.25 mg of bevacizumab at baseline and 6 weeks; Beva II: intravitreal injection of 2.5 mg of bevacizumab at baseline and 6 weeks; Beva III: intravitreal injection of 1.25 mg of bevacizumab at baseline and sham injection at 6 weeks; Beva IV: intravitreal injection of 1.25 mg of bevacizumab at baseline and 6 weeks with photocoagulation at 3 weeks; Beva: bevacizumab; Rani: ranibizumab; Triam: triamcinolone acetonide.

**Table 2:** Overview of the visual acuity outcomes of the Protocol T.

Visual acuity letter score and Snellen equivalent	Aflibercept	Bevacizumab	Ranibizumab	Aflibercept vs. bevacizumab		Aflibercept vs. ranibizumab		Ranibizumab vs. bevacizumab	
				Difference (95% CI)	*p* value^a^	Difference (95% CI)	*p* value^a^	Difference (95% CI)	*p* value^a^
*Letter score of <69, equivalent to 20/50 or worse, at baseline*									
Number of eyes	102	102	101						
Visual acuity at baseline									
Mean (SD) letter score	56.2 (11.1)	56.6 (10.6)	56.5 (9.9)						
Approximate Snellen equivalent	20/80	20/80	20/80						
Visual acuity at 1 year									
Mean (SD) letter score	75.2 (10.9)	68.5 (13.6)	70.7 (12.0)						
Approximate Snellen equivalent	20/32	20/40	20/40						
Change from baseline in letter score									
Mean (SD) improvement	18.9 (11.5)	11.8 (12.0)	14.2 (10.6)	6.5 (2.9 to 10.1)	<0.001	4.7 (1.4 to 8.0)	0.003	1.8 (−1.1 to 4.8)	0.21
Improvement of ≥10 letters, *n* (%)	79 (77)	61 (60)	70 (69)	17 (2 to 31)	0.02	10 (−4 to 23)	0.20	7 (−6 to 20)	0.28
Worsening of ≥10 letters, *n* (%)	1 (1)	4 (4)	2 (2)	−3 (−7 to 2)	0.56	−1 (−5 to 3)	0.56	−1 (−6 to 3)	0.56
Improvement of ≥15 letters, *n* (%)	68 (67)	42 (41)	50 (50)	24 (9 to 39)	<0.001	18 (4 to 32)	0.008	6 (−7 to 19)	0.34
Worsening of ≥15 letters, *n* (%)	1 (1)	2 (2)	2 (2)	0 (−3 to 3)	0.85	−1 (−4 to 2)	0.85	1 (−3 to 4)	0.85

*Letter score of 78 to 69, equivalent to 20/32 to 20/40, at baseline*									
Number of eyes	106	104	105						
Visual acuity at baseline									
Mean (SD) letter score	73.5 (2.6)	72.8 (2.9)	73.4 (2.7)						
Approximate Snellen equivalent	20/32	20/40	20/40						
Visual acuity at 1 year									
Mean (SD) letter score	81.4 (8.3)	79.9 (10.1)	81.6 (6.8)						
Approximate Snellen equivalent	20/25	20/25	20/25						
Change from baseline in letter score									
Mean (SD) improvement	8.0 (7.6)	7.5 (7.4)	8.3 (6.8)	0.7 (−1.3 to 2.7)	0.69	−0.4 (−2.3 to 1.5)	0.69	1.1 (−0.9 to 3.1)	0.69
Improvement of ≥10 letters, *n* (%)	53 (50)	47 (45)	52 (50)	6 (−9 to 21)	0.82	0 (−13 to 14)	0.95	6 (−10 to 21)	0.82
Worsening of ≥10 letters, *n* (%)	4 (4)	2 (2)	1 (1)	2 (−3 to 6)	0.54	3 (−1 to 7)	0.54	−1 (−4 to 2)	0.54
Improvement of ≥15 letters, *n* (%)	19 (18)	17 (16)	16 (15)	2 (−7 to 11)	0.73	4 (−5 to 12)	0.73	−2 (−10 to 7)	0.73
Worsening of ≥15 letters, *n* (%)	2 (2)	1 (1)	1 (1)	1 (−2 to 4)	0.99	1 (−2 to 4)	0.99	0 (−3 to 3)	0.99

^a^Treatment group comparisons were performed with ANCOVA models adjusted for continuous baseline visual acuity or from binomial regression models adjusted for categorical baseline visual acuity (adapted from Diabetic Retinopathy Clinical Research Network et al. [29]). CI: confidence interval; SD: standard deviation; *n* = number.

**Table 3:** Overview of the functional and anatomic results of ranibizumab.

Study	Ref.	Duration (w)	Regimen	*N* (eyes)	BCVA (ETDRS letters)		CRT (*µ*m)	
					Baseline	Change	Baseline	Change
READ-2	[43]	24	Rani	42	24.85	7.24^*∗∗*^	422	−106.3
			Rani + laser	42	24.87	3.8	474.5	−117.2
			Laser	42	28.35	−0.43	439.6	−82.8

RESOLVE	[44]	52	Rani	102	60.2 (9.9)	7.8 (7.7)^*∗∗*^	455.4 (114.2)	−194.2 (135.1)^*∗∗*^
			Sham	49	61.1 (9.0)	−0.1 (9.8)	448.9 (102.8)	−48.4 (153.4)

RESTORE	[45]	52	Rani + sham	116	N.A.	6.1 (6.3)^*∗∗*^	N.A.	−118.7 (115.1)^*∗∗*^
			Rani + laser	118	N.A.	5.9 (7.9)^*∗∗*^	N.A.	−128.3 (114.3)^*∗∗*^
			Laser + sham	111	N.A.	0.8 (8.6)	N.A.	−61.3 (132.3)

RESTORE ^Ext.^	[55]	156	Rani + sham	116	N.A.	8.0 (1.1)	116	−142.1
			Rani + laser	118	N.A.	6.7 (1.1)	118	−145.9
			Laser + sham	111	N.A.	6.0 (1.1)	111	−142.7

DRCR.net	[47]	56	Rani 0.5	113	68 (56–−75)	+2 (−3 to +7)^*∗∗*^	352 (283–−476)	−26 (−92 to +15)^*∗*^
			Triam	109	67 (59–−75)	+1 (−3 to +8)^*∗∗*^	359 (271–−472)	−75 (−168 to −17)^*∗∗*^
			Sham	123	67 (52–−75)	−2 (−8 to +3)	355 (285–−510)	0 (−80 to +70)

RISE	[46]	104	Rani 0.3 mg	125	54.7 (12.6)	12.5^*∗∗∗*^	4745. (174.8)	−250.6^*∗∗∗*^
			Rani 0.5 mg	125	56.9 (11.6)	11.9^*∗∗∗*^	463.8 (144.0)	−253.1^*∗∗∗*^
			Sham	127	57.2 (11.1)	2.6	467.3 (152.0)	−133.4

RIDE	[46]	104	Rani 0.3 mg	125	57.5 (11.6)	10.9^*∗∗∗*^	482.6 (149.3)	−259.8^*∗∗∗*^
			Rani 0.5 mg	127	56.9 (11.8)	12.0^*∗∗∗*^	463.8 (175.5)	−270.7^*∗∗∗*^
			Sham	130	57.3 (11.2)	2.3	447.4 (154.4)	−125.8

RISE	[49]	156	Rani 0.3 mg	125	54.7 (12.6)	14.2 (12.8)^*∗∗∗*^	4745. (174.8)	−261.2 (196.5)
			Rani 0.5 mg	125	56.9 (11.6)	11.0 (12.9)^*∗∗∗*^	463.8 (144.0)	−269.1 (178.9)
			Sham	127	57.2 (11.1)	4.3 (14.9)	467.3 (152.0)	−200.1 (215.6)

RIDE	[49]	156	Rani 0.3 mg	125	57.5 (11.6)	10.6 (12.9)^*∗∗∗*^	482.6 (149.3)	−261.8 (180.8)
			Rani 0.5 mg	127	56.9 (11.8)	11.4 (16.3)^*∗∗∗*^	463.8 (175.5)	−266.7 (207.8)
			Sham	130	57.3 (11.2)	4.7 (13.3)	447.4 (154.4)	−213.2 (193.5)

RISE	[57]	208	Rani 0.3 mg	89	54.4 (12.0)	−1.7 (−3.6 to 0.2)	475.9 (170.2)	23.3 (−7.7 to 54.3)
			Rani 0.5 mg	79	56.5 (10.9)	0.8 (−1.1 to 2.7	476.7 (139.5)	4.2 (−17.1 to 25.4)
			Sham	77	57.8 (10.5)	1.3 (−0.3 to 2.9)	462.8 (141.4)	29.6 (3.4–55.7)

RIDE	[57]	208	Rani 0.3 mg	83	58.3 (11.3)	−0.9 (−3.6 to 1.8)	480.3 (186.9)	46.1 (−2.6 to 94.8
			Rani 0.5 mg	84	56.9 (11.8)	0.6 (−1.2 to 2.4	481.1 (163.2)	44.1 (16.1–72.1)
			Sham	88	57.8 (11.4)	−2.6 (−5.6 to 0.5)	441.3 (146.3)	9.6 (−18.4 to 37.6)

REFINE	[64]	52	Rani 0.5 mg	307	59.6 (10.5)	7.8 (0.7)^*∗∗*^	473.4 (166.1)	−146.5 (157.6)^*∗∗*^
			Laser	77	58.2 (9.4)	2.5 (7.8)	475.0 (161.5)	−85.9 (166.6)

*Note*. READ-2: measured subfoveal thickness. RESOLVE: ranibizumab group included intravitreal injections of 0.3 mg and 0.5 mg (51 eyes each). DRCR.net: patients received laser besides their treatment assigned; measured central subfield thickness; differences were calculated at week 14 (primary outcome of the study). RESTORE extension: all patients enrolled in the extension study were eligible to receive intravitreal ranibizumab 0.5 mg injections (subgroups were maintained for evaluating the effect of ranibizumab according to the initial treatment). ^*∗*^*p* < 0.01 vs. reference comparator (laser, sham, triamcinolone, etc.). ^*∗∗*^*p* < 0.001 vs. reference comparator (laser, sham, triamcinolone, etc.). ^*∗∗∗*^*p* < 0.0001 vs. reference comparator (laser, sham, triamcinolone, etc.). w: weeks; BCVA: best-corrected visual acuity; EDTRS: Early Treatment of Diabetic Retinopathy Study; CRT: central retina thickness; DRCR.net: Diabetic Retinopathy Clinical Research Network; Rani: ranibizumab; Triam: triamcinolone acetonide.

**Table 4:** Overview of the functional and anatomic results of aflibercept.

Study	Ref.	Duration (w)	Regimen	*N* (eyes)	BCVA (ETDRS letters)		CRT (*µ*m)	
					Baseline	Change	Baseline	Change
VISTA	[70]	52	IA4W	154	58.9 (10.8)	12.5^*∗∗∗*^	485 (157)	−185.9^*∗∗∗*^
			IA8W	151	59.4 (10.9)	10.7^*∗∗∗*^	479 (154)	−183.1^*∗∗∗*^
			Laser	154	59.7 (10.9)	0.2	483 (153)	−73.3
	[70]	100	IA4W	154	58.9 (10.8)	11.5^*∗∗∗*^	485 (157)	−191.4^*∗∗∗*^
			IA8W	151	59.4 (10.9)	11.7^*∗∗*^	479 (154)	−191.1^*∗∗*^
			Laser	154	59.7 (10.9)	6.3	483 (153)	−83.9
	[74]	148	IA4W	154	58.9 (10.8)	10.4^*∗∗∗*^	485 (157)	−200.4^*∗∗∗*^
			IA8W	151	59.4 (10.9)	10.5^*∗∗∗*^	479 (154)	−190.1^*∗∗∗*^
			Laser	154	59.7 (10.9)	1.4	483 (153)	−109.8

VIVID	[70]	52	IA4W	136	60.8 (10.7)	10.5^*∗∗∗*^	502 (144)	−195.0^*∗∗∗*^
			IA8W	135	58.8 (11.2)	10.7^*∗∗∗*^	518 (147)	−192.4^*∗∗∗*^
			Laser	132	60.8 (10.6)	1.2	540 (152)	−66.2
	[70]	100	IA4W	136	60.8 (10.7)	11.8^*∗∗*^	502 (144)	211.8^*∗∗∗*^
			IA8W	135	58.8 (11.2)	10.6^*∗∗*^	518 (147)	−195.8^*∗∗∗*^
			Laser	132	60.8 (10.6)	5.5	540 (152)	−85.7
	[74]	148	IA4W	136	60.8 (10.7)	10.3^*∗∗∗*^	502 (144)	215.2^*∗∗∗*^
			IA8W	135	58.8 (11.2)	11.7^*∗∗∗*^	518 (147)	−202.8^*∗∗∗*^
			Laser	132	60.8 (10.6)	1.6	540 (152)	−122.6

Baker et al	[80]	104	IA4W	226	85.2 (3.5)	0.9 (6.4)	306 (55)	−48 (65)
			Laser	240	85.2 (3.8)	0.1 (6.3)	314 (52)	−41 (75)
			Observation	236	85.2 (3.8)	−0.4 (6.4)	314 (64)	−42 (75)

*Note*. ^*∗*^*p* < 0.01 vs. laser. ^*∗∗*^*p* < 0.001 vs. laser. ^*∗∗∗*^*p* < 0.0001 vs. laser. Baker et al. measured central subfoveal thickness. w: weeks; BCVA: best-corrected visual acuity; EDTRS: Early Treatment of Diabetic Retinopathy Study; CRT: central retina thickness; IA4W: intravitreal injections of aflibercept every 4 weeks; IA8W: intravitreal injections of aflibercept every 8 weeks.

**Table 5:** Overview of the functional and anatomic results of dexamethasone intravitreal (DEX) and fluocinolone intravitreal implants.

Study	Ref.	Duration (m)	Regimen	*N* (eyes)	BCVA (ETDRS letters)		CRT (*µ*m)	
					Baseline	Change	Baseline	Change
MEAD	[100]	36	DEX 0.35 mg	351	56.1 (9.9)	18.4%^1^^*∗∗∗*^	463.0 (157.1)	−107.9 (135.8)^*∗∗∗*^
			DEX 0.7 mg	347	55.5 (9.7)	22.2%^1^^*∗∗*^	466.8 (159.5)	−111.6 (134.1)^*∗∗∗*^
			Sham	350	56.9 (8.7)	12.0%^1^	460.9 (132.6)	−41.9 (116.0)

BEBORDEX	[104]	12	DEX 0.7 mg	46	55.5 (12.5)	7.9 (11.6)	474.3 ± 95.9	−179.0 (88.8)^*∗∗*^
			Beva 0.5 mg	42	56.3 (11.9)	7.5 (11.0)	503 ± 140.9	−93.0 (131.6)
	[105]^†^	24	DEX 0.7 mg	46	55.5 (12.5)	6.9 (2.7 to 11.1)	474.3 ± 95.9	N.A.
			Beva 0.5 mg	42	56.3 (11.9)	9.6 (6.9 to 12.3)	503 ± 140.9	N.A.

Shah et al	[107]	7	DEX 0.7 mg	27	59 (12)	5.8 (7.6)	458 (100)	−122 (95)^*∗∗∗*^
			Beva 0.5 mg	23	59 (13)	5.6 (6.1)	485 (122)	−14 (141)

Maturi et al	[108]	6	DEX + Rani	65	63 (12)	2.7 (9.8)	375 (97)	−110 (86)^*∗∗∗*^
			Sham + Rani	64	63 (13)	3.0 (7.1)	396 8122)	−62 (97)

FAME	[132]	24	IFSR 0.2 *µ*g	375	53.3 (12.7)	*p* = 0.019	460.8 (160.0)	*p* ≤ 0.003
			IFSR 0.5 *µ*g	393	52.9 (12.2)	*p* = 0.015	485.1 (173.8)	*p* ≤ 0.003
			Sham	185	54.7 (11.3)	—	451.3 (152.0)	—

FAME^‡^	[133]	36	IFSR 0.2 *µ*g	165	54.7 (11.7)	2.4	466.6 (152.9)	−173.1^*∗*^
			Sham	72	557. (11.5)	2.3	435.0 (149.1)	−115.6

FAME^‡‡^	[133]	36	IFSR 0.2 *µ*g	209	52.2 (13.4)	7.6^*∗∗*^	456.2 (165.9)	−186.8
			Sham	112	54.0 (11.5)	1.8	461.8 (153.5)	−160.0

*Note.* ^*∗*^ *p* < 0.05 vs. comparator/sham. ^*∗∗*^*p* < 0.01 vs. comparator/sham. ^*∗∗∗*^*p* < 0.001 vs. comparator/sham. ^*∗∗∗∗*^*p* < 0.0001 vs. comparator/sham. ^1^Proportion of patients with a ≥ 15-letter improvement in best-corrected visual acuity (BCVA) from baseline at the year 3. ^†^For those eyes that were pseudophakic at baseline, the mean improvement in BCVA was 8.9 letters (95% confidence interval (CI), 2.0–13.4) for those treated with the dexamethasone (DEX) implant and 7.7 letters (95% CI, 3.03–14.8) for those treated with bevacizumab; *p* = 0.77. For the eyes that were phakic at baseline, the mean improvement in BCVA was 5.8 letters (95% CI, 0.07–11.5) for those treated with the DEX implant and 10.2 letters (95% CI, 7.17–13.3) for those treated with bevacizumab; *p* = 0.19. The specific data regarding central retinal thickness of BEVORDEX study at 24 months are not available from the literature [105] and hence are not listed in this table. Shah et al. [107] measured central subfoveal thickness. Maturi et al. [108] measured central subfoveal thickness. FAME: The specific data regarding BCVA and central retinal thickness are not available from the literature [112] and hence are not listed in this table. The *p* value corresponded to the difference between intravitreal fluocinolone sustained-release (IFSR) and sham. ^‡^Nonchronic diabetic macular edema (DME) (<3 years). ^‡‡^Chronic DME (≥3 years). m: months; BCVA: best-corrected visual acuity; EDTRS: Early Treatment of Diabetic Retinopathy Study; CRT: central retina thickness; DEX: dexamethasone implant; Beva: bevacizumab; IFSR: intravitreal fluocinolone sustained-release.

## Data Availability

No data were used to support the findings of this study.
